# Regenerative neurogenesis: the integration of developmental, physiological and immune signals

**DOI:** 10.1242/dev.199907

**Published:** 2022-05-03

**Authors:** Thomas Becker, Catherina G. Becker

**Affiliations:** 1 Center for Regenerative Therapies at the TU Dresden, Technische Universität Dresden, 01307 Dresden, Germany; 2 Centre for Discovery Brain Sciences, University of Edinburgh Medical School, Biomedical Science, Edinburgh, EH16 4SB, Scotland

**Keywords:** Müller cells, Cytokines, Ependymo-radial glia, Macrophages, Neurotransmitters, Radial glia

## Abstract

In fishes and salamanders, but not mammals, neural stem cells switch back to neurogenesis after injury. The signalling environment of neural stem cells is strongly altered by the presence of damaged cells and an influx of immune, as well as other, cells. Here, we summarise our recently expanded knowledge of developmental, physiological and immune signals that act on neural stem cells in the zebrafish central nervous system to directly, or indirectly, influence their neurogenic state. These signals act on several intracellular pathways, which leads to changes in chromatin accessibility and gene expression, ultimately resulting in regenerative neurogenesis. Translational approaches in non-regenerating mammals indicate that central nervous system stem cells can be reprogrammed for neurogenesis. Understanding signalling mechanisms in naturally regenerating species show the path to experimentally promoting neurogenesis in mammals.

## Abstract (precis)

**Summary:** This Review summarises the array of signals that influence regenerative neurogenesis in the central nervous system of zebrafish, indicating possible avenues for activation of similar signalling pathways in non-regenerating mammals.

## Introduction

**Fig. 1.**
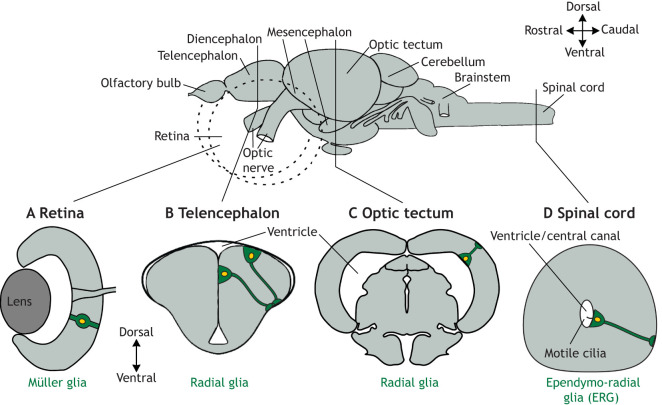
**Stem/progenitor cells capable of injury-induced neurogenesis are distributed over the zebrafish CNS.** An overview of the adult zebrafish CNS (retinae indicated by dashed lines) and schematic cross-sections at the indicated levels, retina (A), telencephalon (B), optic tectum (C) and spinal cord (D), illustrate stem/progenitor cells with regenerative potential. Note that stem/progenitor cells (shown in green) span the width of the neural tissue and have ventricular contact in most CNS regions. In the telencephalon, the somata of radial glia face the outside of the brain owing to eversion of the tissue during development (Folgueira et al., 2012).

Fishes and salamanders (anamniotes) have a high capacity to regenerate neurons after injury to the central nervous system (CNS) (Joven et al., 2019; Lange and Brand, 2020). In mammals, however, this capacity is very limited (Becker et al., 2018; Diotel et al., 2020). Neural stem or progenitor cells in fishes are likely to also have physiological functions in neurotransmitter clearance and ion homeostasis that are similar to those of parenchymal astrocytes in mammals, as indicated by the expression of genes involved in these processes, such as *excitatory amino acid transporter 2* (*eaat2*; *slc1a2*) and *aquaporin 4* (*aqp4*). Neural stem/progenitor cells also express the well-known astrocytic marker glial fibrillary acidic protein (Gfap) in zebrafish (Jurisch-Yaksi et al., 2020). Dedicated astrocytes are rare in zebrafish (Chen et al., 2020). In addition, neural stem/progenitor cells in fishes have a high capacity to generate neurons after injury. These stem/progenitor cells are called Müller glia in the retina, radial glia in the brain (i.e. in the telencephalon and optic tectum) and ependymo-radial glia (ERG) in the spinal cord (Becker and Becker, 2015; Lenkowski and Raymond, 2014; Lindsey et al., 2018) (Fig. 1). All these cells contact the ventricle and are probably direct descendants of neuroepithelial cells that produce neurons during development. Similar astrocyte-like or ependymal cells exist in mammals and are triggered to proliferate after injury, but mostly undergo gliogenesis and thus produce scar tissue (Grégoire et al., 2015; Stenudd et al., 2015). This divergence between species makes it interesting to learn how stem/progenitor cells in anamniotes can be activated and driven to undergo a cell-fate switch to produce neurons after injury. Recently, accumulating evidence has indicated that stem/progenitor cells are exposed to a bewildering array of signals after injury. Perhaps unsurprisingly, these are composed of re-expressed developmental growth factors, but also physiological signals (such as neurotransmitters) and regeneration-specific signals from infiltrating immune cells, which the stem/progenitor cells may not have been exposed to during embryonic development. These signals are integrated by the stem/progenitor cells via a variety of signal transduction pathways involved in trophic factor, cytokine and neurotransmitter signalling to converge on altering the state of the cells to generate neurons. Here, we focus on progress in zebrafish CNS regeneration, because this model organism has a high capacity for regeneration and is genetically tractable, as well as being used for pharmacological and genetic CNS regeneration screening (Chapela et al., 2019; Keatinge et al., 2021). Research in zebrafish has revealed several endogenous signals that activate the regeneration of new neurons. We first describe regeneration paradigms in the retina, brain and spinal cord. We then compare signals and downstream signalling between different CNS regions. Finally, we discuss how these inform pro-regenerative approaches in non-regenerating mammals.

## CNS regeneration paradigms in zebrafish

All the components of the zebrafish CNS, including the retina, different regions of the brain and the spinal cord (Fig. 1), show different levels of constitutive neurogenesis during homeostasis, which may change in response to injury. Here, we compare these regions in the context of specific signalling molecules.

### Retina

The retina develops from a sheet of neural epithelium; different cell layers delaminate from this sheet in a specific sequence, with retinal ganglion cells first and Müller cells last to generate the laminated structure of the vertebrate retina (Stenkamp, 2015) (Fig. 1A).

In the uninjured retina, Müller glia mainly generate a few rod photoreceptor precursor cells (Bernardos et al., 2007). However, after injury, Müller cells become highly proliferative and produce new neurons in zebrafish, but not in mammals (Lahne et al., 2020). Interestingly, in salamanders, it is not the Müller glia but pigment epithelium cells that form the stem/progenitor cell population that gives rise to new neurons after injury (Islam et al., 2014).

Different injury paradigms have been used to study regeneration in the zebrafish retina, including mechanical stab injury (Sharma and Ramachandran, 2019), light injury of photoreceptors (Thomas et al., 2012) and neurotoxin injections into the eye (Powell et al., 2016). Additionally, specific cell types (e.g. rod photoreceptors) can be ablated by transgenically overexpressing the bacterial enzyme nitroreductase, which converts a pro-drug into a cytotoxic compound only in the cells that express the enzyme (White et al., 2017). In all cases, zebrafish Müller glia produce all retinal neuron types, including those that have been ablated (Fausett and Goldman, 2006; Fimbel et al., 2007; Powell et al., 2016; Vihtelic and Hyde, 2000).

### Brain

The vertebrate brain develops from the neural tube and is patterned along two principal axes: the dorsoventral axis is patterned by morphogen gradients from the dorsal-most roof plate cells and ventral-most floor plate cells. Dorsal signals include Wnt and bone morphogenetic proteins (Bmps), whereas sonic hedgehog (Shh) is the major ventral morphogen. These opposing gradients set up ventricular progenitor cell domains, which in turn generate specific neuronal cell types. Patterning along the rostrocaudal axis is controlled by Wnts and retinoic acid (Altmann and Brivanlou, 2001; Leung and Shimeld, 2019; Lupo et al., 2006).

These mechanisms contribute to setting up the typical brain anatomy in vertebrates, including in zebrafish. From rostral to caudal, the telencephalon, diencephalon, mesencephalon, cerebellum and hindbrain form separate functional units (Fig. 1). The central lumen of the former neural tube transforms into ventricles. The wall of the former neural tube forms the brain regions that are limited towards the ventricles by the ventricular or ependymal cell layer. The outer surface is limited by the pia mater and therefore called the pial surface (Nieuwenhuys et al., 1998).

In the adult zebrafish brain, *gfap*-expressing stem/progenitor cells with ventricular somata and radial processes contact the pial surface with endfeet. These cells are often called ‘radial glial cells’ but should not be confused with the transient cell type of the same name in the developing mammalian cortex (Villalba et al., 2021) (Fig. 1B,C). Radial glia in zebrafish are likely direct descendants of ventricular cells during development and increase proliferation and neurogenesis after brain injury (Barbosa et al., 2015; Kyritsis et al., 2012; Lindsey et al., 2019; Ueda et al., 2018). In the cerebellum, however, neuroepithelial cells produce new neurons (Kaslin et al., 2017). Local and regional differences in radial glial cells probably exist but require further exploration. For example, in the diencephalon, radial glial cells are positive for either Gfap, Olig2 or both. They show differences in their proliferative response to the ablation of dopaminergic neurons, which might signify different classes of progenitor cells (Caldwell et al., 2019).

Of note, the adult zebrafish brain contains several domains of continuous neurogenesis, some of which may be comparable to those in mammals in the subventricular zone and the hippocampus (Adolf et al., 2006; Chapouton et al., 2006). Neurogenic domains are more frequent in the brain of zebrafish compared with mammals and have been reviewed in detail elsewhere (Diotel et al., 2020; Lange and Brand, 2020). Areas of constitutive neurogenesis may be primed for regenerating neurons after injury and there are indications from the dopaminergic system that dopaminergic nuclei with ongoing neurogenesis show more efficient neuronal replacement after ablation than those with little detectable turnover in uninjured animals (Caldwell et al., 2019). Interestingly, ongoing constitutive neurogenesis can be sufficient to replace dopaminergic neurons in some brain areas, so does not require an injury signal (Godoy et al., 2015; McPherson et al., 2016).

In the zebrafish brain, injuries are often performed by stabbing the telencephalon through a nostril (Baumgart et al., 2012; Grandel et al., 2006) or injecting excitotoxins, such as quinolinic acid, into the brain (Skaggs et al., 2014). Stab injuries are also used for the optic tectum or cerebellum (Kaslin et al., 2017; Ueda et al., 2018). In addition, intraventricular injection of the amyloid-β42 neurotoxic peptide is also used to inflict neuronal damage and induce stem/progenitor cell activation (Bhattarai et al., 2016). Individual cell types can also be ablated; for example, dopaminergic neurons can be specifically ablated with the toxin 6-hydroxy-dopamine (Caldwell et al., 2019; Godoy et al., 2015).

### Spinal cord

Spinal cord patterning along the principal axes is comparable to that of the brain described above; however, the portion of the spinal cord caudal to segments 8-12 is generated from bi-potent neuromesodermal progenitors (Gouti et al., 2014; Martin and Kimelman, 2008). The different origin of the caudal spinal cord, and its stem/progenitor cells, could potentially also entail divergent mechanisms of regenerative neurogenesis compared with other CNS regions (see below).

ERG remain at the ventricle, possess one to two motile cilia and project their radial processes to the pial surface (Fig. 1D) (Becker and Becker, 2015; Ribeiro et al., 2017). These cells are heterogeneous; they retain their dorsoventral progenitor-domain identity from development, but do not produce new neurons in the uninjured spinal cord (Reimer et al., 2008). For example, a ventrolateral domain of ERG express *olig2* in adult zebrafish and is, therefore, the adult equivalent of the motor neuron progenitor domain seen during development in all vertebrates (Ligon et al., 2006).

After injury, *olig2*-expressing ERG in the ventrolateral domain and adjacent domains below and above start proliferating and produce different cell types at different dorsoventral positions of the spinal cord, suggesting that progenitor zones are fate-restricted and are able to regenerate the neuronal cell types they produce during embryonic development (Kuscha et al., 2012). In addition to producing neurons, some ERG undergo epithelial-to-mesenchymal transition to generate elongated astrocyte-like cells that re-connect the injured spinal cord (Klatt Shaw et al., 2021).

The adult spinal cord is usually lesioned by mechanical transection or crush injury (Goldshmit et al., 2012; Hui et al., 2014; Mokalled et al., 2016; Reimer et al., 2008), but transgenic cell ablation has also been used (Ohnmacht et al., 2016). Of note, spinal cord injury in larvae presents a very rapid experimental paradigm that also leads to lesion-induced neurogenesis (Briona and Dorsky, 2014; Cavone et al., 2021). Moreover, larval developmental stages are amenable to video observations, which are challenging to perform in adults (Vandestadt et al., 2021; Wehner et al., 2017). Keep in mind, however, that during larval and juvenile growth, new neurons are constantly produced. Therefore, these stages may be more prone to regeneration by just increasing ongoing neurogenesis. Adult zebrafish (>4 months) display hardly any neurogenesis in the spinal cord (Reimer et al., 2008), a phenomenon that differentiates the adult spinal cord from the brain and retina, and makes it more comparable to its mammalian counterpart.

## Developmental signalling

If stem/progenitor cells in the adult CNS are descendants of neuroepithelial cells during development, it is plausible that adult stem/progenitor cells would be sensitive to the same signals after an injury that promote developmental neurogenesis from neuroepithelial cells. In this section, we review several signalling mechanisms that are important for CNS development and regeneration after injury.

### Signalling pathways

#### Sonic hedgehog signalling

**Fig. 2.**
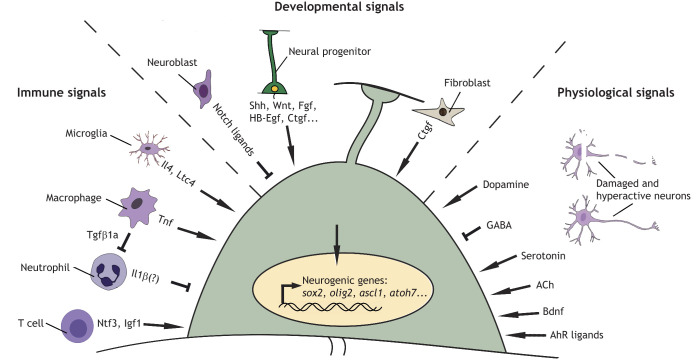
**The signalling environment of neural stem/progenitor cells is complex in regenerative neurogenesis.** The potential signalling environment of an idealised neural stem/progenitor cell, composed of what is known for Müller cells, radial glia and ependymo-radial glia (green). Different signals can be categorised into immune system-derived signals (left), re-deployed developmental signals (centre) and physiological signals (right), as divided by the dashed lines. These signals may synergise to lead to changes in epigenetic chromatin modifications and reactivation of neurogenic gene expression programmes (nucleus; yellow). Immune cells that arrive at the injury site (neutrophils, microglia, macrophages and T cells) signal with specific cytokines, growth factors and other signalling molecules to stem/progenitor cells after injury. Note that immune cells extensively interact with each other; here, only one such interaction is shown: macrophages controlling neutrophils via Tgfβ1. Developmental signals that are re-deployed are mostly released by stem/progenitor cells themselves and differentiating neurons in an auto- or paracrine fashion, but may also be released from non-neural cell types, such as fibroblasts. Lastly, physiological signals, such as neurotransmitters, are derived from stressed or dying neurons. For abbreviations see text and Table 1.

The classical developmental morphogen signal Shh promotes regenerative neurogenesis across the retina, midbrain and spinal cord (Table 1; Fig. 2). Pharmacological stimulation and inhibition of the Shh pathway increases or reduces neurogenesis, respectively. Moreover, changes in expression of Shh target genes in stem/progenitor cells (e.g. *ptch2*) suggest that Shh signalling acts directly on stem/progenitor cells (Reimer et al., 2009; Thomas et al., 2018; Ueda et al., 2018).

**Table 1.**
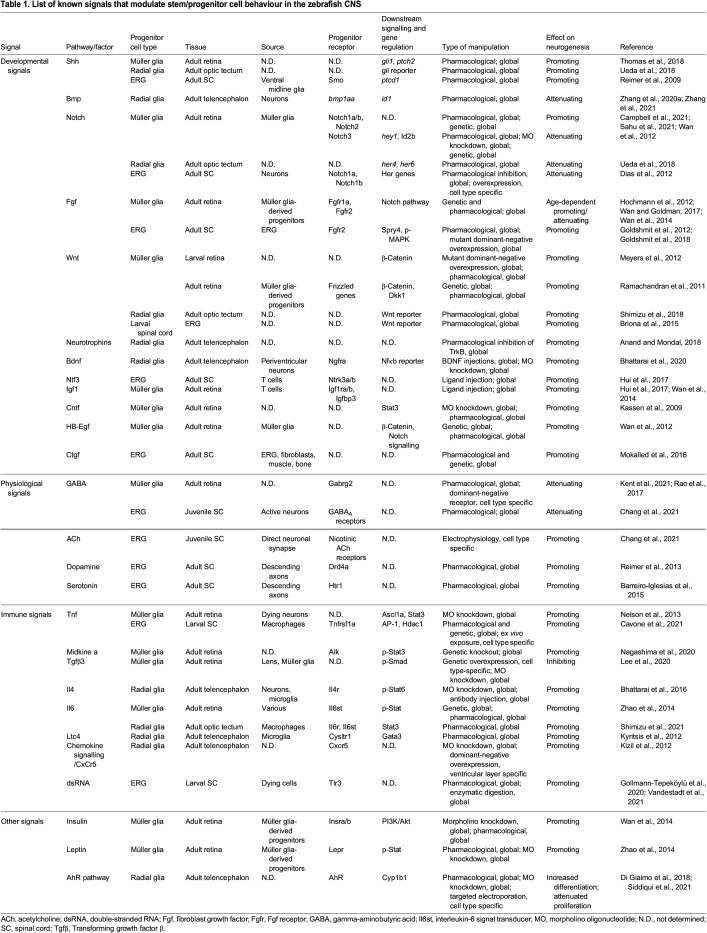
List of known signals that modulate stem/progenitor cell behaviour in the zebrafish CNS

#### Wnt signalling

Similarly, Wnt signalling promotes regenerative neurogenesis in the retina (Meyers et al., 2012; Ramachandran et al., 2011), optic tectum (Shimizu et al., 2018) and spinal cord (Briona et al., 2015). Changes in pathway activity after injury are shown by changes in Wnt reporter activity in the stem/progenitor cells, and Wnt signalling activates canonical β-catenin signalling in most cases (Meyers et al., 2012; Ramachandran et al., 2011; Shimizu et al., 2018), but not all (Briona et al., 2015; Lindsey et al., 2019).

#### Bmp signalling

In the telencephalon of adult zebrafish, components of the Bmp signalling pathway are upregulated after stab injury. Bmp signalling leads to upregulation of the transcription factor Id1, which inhibits neurogenesis in the telencephalon. This suggests that Bmp is a negative regeneration signal (Zhang et al., 2020a, 2021).

#### Notch signalling

Notch signalling maintains the progenitor cell state during development and the pathway's role in regeneration has been reviewed in some detail (Alunni and Bally-Cuif, 2016; Campbell et al., 2022). Although global pharmacological inhibition or genetic activation of Notch signalling indicates an attenuating role for Notch signalling in neurogenesis (Dias et al., 2012; Ueda et al., 2018), Notch3 seems to be a quiescence-inducing receptor in zebrafish retinal radial glia, whereas Notch1 and Notch2 signalling mediate neuronal differentiation (Campbell et al., 2021; Sahu et al., 2021). These functions are also observed during ongoing constitutive neurogenesis in the adult telencephalon (Alunni et al., 2013; Than-Trong et al., 2018). This discrepant role of Notch receptors can be understood as a balancing act between stem/progenitor cell renewal and neuronal differentiation, resulting in the maintenance of the stem/progenitor cell pool. At the molecular level, Notch signalling leads to increased expression of Her effector genes in stem/progenitor cells (Campbell et al., 2021; Sahu et al., 2021; Ueda et al., 2018).

#### Fgf signalling

Fgf signalling promotes regeneration in the retina (Hochmann et al., 2012; Wan and Goldman, 2017; Wan et al., 2014) and spinal cord (Goldshmit et al., 2012). Fgf signalling acts through MAPK to activate canonical downstream genes (e.g. *spry4*) after spinal injury (Goldshmit et al., 2012, 2018). Notably, the specific signalling molecules and receptors may differ from development (Goldshmit et al., 2018), indicating that regeneration is not a simple recapitulation of developmental programmes. Supporting the idea of regeneration-specific gene regulation is the observation that *lin28*, which encodes an RNA-binding protein, is upregulated in Muller glia-derived progenitors in the adult injured retina, but not expressed in progenitors in the developing retina at 24 h post-fertilisation (Ramachandran et al., 2010).

#### Tgfβ signalling

Tgfβ signalling has been extensively studied in retinal regeneration and its role is complex. Studies using indirect manipulation and gene expression analysis have suggested positive roles for the pathway (Conedera et al., 2021; Lenkowski and Raymond, 2014). A study using pharmacological inhibition and injection of human TGFβ1 into the retina has shown a biphasic role for Tgfβ signalling in promoting proliferation in the early phase after retinal injury and a late role in ending the proliferation of Müller glial cells (Sharma et al., 2020). Finally, a recent study has shown that the pathway is active in quiescent Müller glia cells and not in proliferating Müller glia-derived progenitors, as measured by phospho-Smad (p-Smad) levels. Morpholino knockdown and Müller glia-specific overexpression experiments have shown that Tgfβ3 inhibits Müller glia proliferation. Furthermore, interactions with the Notch pathway have led to the speculation that Tgfβ3 may be involved in preserving the ‘stemness’ of Müller glia (Lee et al., 2020).

#### Neurotrophin signalling

Manipulations of neurotrophin signalling, such as brain-derived neurotrophic factor (Bdnf) and the neurotrophin receptor TrkB, in the lesioned telencephalon have indicated that they promote regenerative neurogenesis (Anand and Mondal, 2018; Bhattarai et al., 2020). Neurotrophic factors, such as Bdnf and Neurotrophic factor 3 (Ntf3) likely act through TrkB receptor signalling (Anand and Mondal, 2018). Hence, developmental signals appear to activate similar downstream signalling pathways during regeneration as during development. Ciliary neurotrophic factor (Cntf) (Kassen et al., 2009), and heparin-binding EGF-like growth factor (HB-Egf) (Wan et al., 2012) also promote retinal regeneration. In addition, Connective tissue growth factor (Ctgf), which is upregulated by glial cells and non-neural cells after injury, promotes regenerative neurogenesis in the adult spinal cord (Mokalled et al., 2016).

#### Primary cilia

Many of these trophic and morphogen signals are detected by the so-called primary (non-motile) cilia during vertebrate CNS development (Hasenpusch-Theil and Theil, 2021). Stem/progenitor cells in the brain (Ogino et al., 2016) and spinal cord (Ribeiro et al., 2017), but not in the optic tectum (Corbo and Fulop, 2020), possess one or two motile cilia (D'Gama et al., 2021). Whether and how these contribute to signal sensing in regeneration needs further investigation.

## Physiological signals

### Neurotransmitters in developmental neurogenesis

In vertebrates, neural stem cells are also exquisitely sensitive to neurotransmitter signalling in different CNS regions (Káradóttir and Kuo, 2018) (Fig. 2). Such sensitivity may enable the brain to match neuron production to developmental or physiological demands. For example, descending dopaminergic axons promote motor neuron generation in the developing spinal cord of zebrafish, which could match the development of descending projections with the maturation of their spinal target (Reimer et al., 2013). However, dopamine levels in the midbrain of salamanders provide negative feedback for their own production there (Berg et al., 2011). Consequently, selective ablation of dopaminergic cells leads to accelerated neurogenesis of this neuronal cell type in the salamander midbrain. In the developing zebrafish spinal cord, serotonin has similar promoting action on neurogenesis as dopamine (Barreiro-Iglesias et al., 2015). In juvenile zebrafish (8-10 weeks of age), exercise increases spinal neurogenesis via acetylcholine, whereas the inhibitory transmitter GABA attenuates spinal neurogenesis (Chang et al., 2021).

### Neurotransmitters in regenerative neurogenesis

After spinal injury, the above neurotransmitters are present and reprise their role as promoters (dopamine, serotonin and acetylcholine) or inhibitors (GABA) of neurogenesis (Table 1; Fig. 2). A reduction in GABA_A_ receptor levels after training enhances neurogenesis and may also be the mechanism by which regenerative neurogenesis is facilitated (Chang et al., 2021). GABA also inhibits regenerative neurogenesis in the retina, where GABA levels are reduced after injury (Kent et al., 2021; Rao et al., 2017). Hence, changes in neurotransmitter abundance after injury promote regenerative neurogenesis by either releasing inhibition or directly promoting neurogenesis.

Relatively little is known about how neurotransmitter signalling acts inside the stem/progenitor cells. Canonical neurotransmitter signalling acts through small G proteins to influence intracellular cyclic adenosine monophosphate (cAMP) levels, which in turn influence other signalling pathways (Berg et al., 2013). For example, pharmacologically increasing dopamine signalling increases expression of the Shh downstream gene *ptch2* concomitantly with regeneration, suggesting that dopamine signalling converges on the Shh pathway, likely through the cAMP/protein kinase A (PKA) pathway (Reimer et al., 2013).

Finally, the aryl hydrocarbon receptor (AhR), a transcription factor, can be activated by kynurenic acid in the injured telencephalon of adult zebrafish and promotes the direct conversion of radial glial cells into neurons (Di Giaimo et al., 2018; Siddiqui et al., 2021).

## Immune signals

Stem/progenitor cells are also sensitive to signals from the immune system. Immune cell-derived signals are not normally part of developmental neurogenesis and thus may play regeneration-specific roles, differentiating immune signals from the developmental and physiological signals described above. Global manipulations of the immune response indicate a positive influence on regenerative neurogenesis; dampening the immune response with the glucocorticoid dexamethasone decreases regenerative neurogenesis. Conversely, enhancing the immune response by treatment with bacterial lipopolysaccharides or the yeast surface glucan zymosan promotes regeneration in the brain (Caldwell et al., 2019; Kyritsis et al., 2012) and spinal cord (Ohnmacht et al., 2016).

The earliest potential signalling molecules that act on progenitor cells and are related to the immune response may come from ‘damage-associated molecular patterns’ (DAMPs) from destroyed cells that lead to a rapid invasion of an injury site by neutrophils. In the spinal cord, for example, neutrophil invasion is followed by the arrival of microglial cells and macrophages (Hui et al., 2010; Tsarouchas et al., 2018). T cells arrive at a CNS injury site last (Hui et al., 2017). However, neutrophils and peripheral macrophages do not invade the retina after selective rod ablation (White et al., 2017), indicating that the immune response may differ for different CNS regions and types of injury. We discuss the role of these molecular and cellular players of the immune system roughly in the order of their appearance (Table 1; Fig. 2).

### DAMPs and neutrophils

DAMPs are composed of double-stranded RNAs released from destroyed cells. DAMPs might activate Toll-like receptors and can act directly on spinal stem/progenitor cells (Vandestadt et al., 2021; Gollmann-Tepeköylü et al., 2020). Enzymatic digestion of free RNAs impairs proliferation of stem/progenitor cells after spinal injury in larval zebrafish. However, mimicking double-stranded RNAs by treatment with synthetic RNAs (polyinosinic-polycytidylic acid) does not augment the proliferation of stem/progenitor cells (Vandestadt et al., 2021). Together, these studies show that tissue damage can signal directly to stem/progenitor cells via double-stranded RNAs that are necessary, but not sufficient, for the proliferative response.

DAMPs also attract invading neutrophils, which in turn release cytokines that may act on stem/progenitor cells. Although a direct action of neutrophils on stem/progenitor cells has not been shown in regenerative neurogenesis, these cells express high levels of the pro-inflammatory cytokine Il1β, which inhibits axonal regeneration after spinal injury on larval zebrafish (Tsarouchas et al., 2018).

### Macrophages

Following neutrophils, invading macrophages secrete several cytokines at sites of spinal injury (Keatinge et al., 2021; Tsarouchas et al., 2018). For example, tumour necrosis factor (Tnf) is released by a pro-regenerative sub-population of invading macrophages after spinal injury in larval zebrafish and directly promotes spinal neurogenesis via the AP-1 signalling complex, which, in turn, alters expression levels of the histone deacetylase *hdac1* necessary for neurogenesis (see below) (Cavone et al., 2021). In the adult zebrafish retina, Tnf derived from dying neurons promotes retinal regeneration (Nelson et al., 2013).

Several cytokine and related signals likely act through the JAK/Stat pathway as indicated by changes in expression or phosphorylation state of Stat3. JAK/Stat signalling has been shown to integrate diverse signals, such as Cntf, Tnf, HB-Egf, Midkine a, insulin and leptin (Nagashima et al., 2020; Nelson et al., 2013; Shimizu et al., 2021; Wan et al., 2014; Zhao et al., 2014).

### Microglia

Tissue-resident macrophages in the brain, called microglia, are the main immune cell type to react to injury (Bosak et al., 2018; reviewed by Mehl et al., 2022). In the injured telencephalon of adult zebrafish, microglia-derived leukotriene C4 (Ltc4) and interleukin 4 (Il4) enhance neuronal regeneration after stab injury and amyloid-β-42 injection, respectively (Bhattarai et al., 2016; Kyritsis et al., 2012).

### T cells

Following the innate immune response, regulatory T cells invade the injury site. Notably, depending on the damaged tissue, these cells release different growth factors. In the spinal cord, T cells release neurotrophin 3 (Ntf3), which promotes neurogenesis in the developing CNS (Averbuch-Heller et al., 1994). Interestingly, T cells invading the damaged retina release insulin-like growth factor 1 (Igf1), which promotes regenerative neurogenesis (Hui et al., 2017). Hence, stem/progenitor cells in the CNS react to several immune-related signals that are modified by the injury environment in a temporal sequence after injury.

## Complex signal integration

The examples introduced above show an array of signals that may act on stem/progenitor cells and these signals overlap in time. To begin to understand how progenitor cells integrate all of these different signals, it is important to appreciate whether effects are direct (i.e. progenitor cells have the necessary receptors and these are activated) or indirect.

### Direct versus indirect signalling

Immune signals act on several different cell types, which could in turn signal to stem/progenitor cells. For example, invading macrophages signal to spinal stem/progenitor cells, but they are also responsible for mitigating the neutrophil response to injury (Houseright et al., 2021). When macrophages are absent (e.g. in *irf8* mutants) or their function is impaired (e.g. by mutating *tgfb1a*), neutrophils linger at the spinal injury site, which impairs regenerative neurogenesis (Keatinge et al., 2021; Tsarouchas et al., 2018). Resolution of the neutrophil reaction also supports regeneration in other organ systems. For example, in the larval heart, transient treatment with a Cdk9 inhibitor accelerates reverse migration of neutrophils and subsequent polarisation of macrophages towards a pro-regenerative phenotype (Kaveh et al., 2021; reviewed by Simões and Riley, 2022). Hence, any global treatments that interfere with macrophage function may indirectly influence stem/progenitor cell behaviour because neutrophil abundance or activation state is changed. Other cell types that react to an injury, such as vascular cells (Dhakal et al., 2021; Fang et al., 2014; Liu et al., 2016) and invading fibroblasts (Tsata et al., 2021), are also likely to provide signals that condition the environment of the proliferating progenitors.

However, for many potential signal/receptor interactions, stem/progenitor cells express appropriate receptors and activate downstream genes or specific pathway reporters (Table 1). Furthermore, stem/progenitor cells are close in proximity to the signal sources, suggesting that, at least in part, these signals act directly on the stem/progenitor cells themselves.

### Crosstalk between signalling pathways

The diversity of signalling pathways needs to be integrated by the stem/progenitor cells and there is evidence for crosstalk between the pathways described above. For example, in the retina, Fgf8a inhibits the Notch pathway in young animals (<3 months) and thus promotes Müller glia proliferation. However, in older animals (>3 months) Fgf8 signalling augments Notch signalling and therefore inhibits neurogenesis (Wan and Goldman, 2017). Interestingly, Tgfβ3 signalling increases Notch signalling in Müller cells (Lee et al., 2020). Notch pathway crosstalk in the injured retina is discussed elsewhere (Campbell et al., 2022). These examples show that different signals may converge onto the same intracellular signalling pathways. Moreover, how these intracellular pathways are affected is also determined by the internal stage of the stem/progenitor cells at different ages of the animals.

As stem/progenitor cells are sensitive to many signals, the temporal coincidence of signals is also important, because these signals can lead to synergistic effects. For example, the experimental addition of leptin and Il11 synergize in stimulating Müller cell proliferation – even at concentrations that have little effect when provided alone (Zhao et al., 2014). Similar synergies exist between Igf1 and Fgf, as well as HB-Egf and Il11, to promote neuroblast formation from Müller cells in the retina (Wan et al., 2014). These data suggest that there is a ‘core set’ of intracellular signalling pathways that govern retinal regeneration. It will be interesting to determine whether this is also the case for other CNS regions.

With the multitude of signals that act on stem/progenitor cells, it is difficult to determine what might trigger regeneration; some signals can stimulate proliferation and/or neurogenesis in the absence of an injury. Intriguingly, in the uninjured telencephalon, where Notch signalling is constitutively active, Notch inhibition alone is sufficient to induce radial glial cell proliferation (Alunni et al., 2013; Chapouton et al., 2010; Dray et al., 2021). Similarly, in the retina, Notch is constitutively active and inhibition of the pathway induces limited Müller glia proliferation (Campbell et al., 2021; Conner et al., 2014), but requires simultaneous manipulations of other signalling pathways for a strong proliferative response (Elsaeidi et al., 2018; Wan and Goldman, 2017). In contrast, in the unlesioned spinal cord, no evidence has been found for activity of the Notch pathway in the absence of injury nor does inhibition of the pathway have any effect (Dias et al., 2012). This difference underscores the presence of regional variation in stem/progenitor cell types.

Similarly, inhibition of GABA receptors (Kent et al., 2021), inhibition of Gsk3β (Ramachandran et al., 2011), Cntf injections (Kassen et al., 2009) or HB-Egf injections (Wan et al., 2012) are all sufficient to elicit at least a proliferation response in adult retinal Müller cells. Furthermore, purmorphamine (a Shh agonist) stimulates the proliferation of radial glia in the uninjured optic tectum. Interestingly, purmorphamine also inhibits neuronal differentiation (Ueda et al., 2018), indicating that stem/progenitor cell proliferation and neuronal differentiation are separate events that are independently regulated.

Non-specific stimulation of the immune system in the absence of an injury increases stem/progenitor cell proliferation and/or neurogenesis in the brain: i.e. in the telencephalon, diencephalon, optic tectum and cerebellum (Caldwell et al., 2019; Kyritsis et al., 2012; Ueda et al., 2018). In the optic tectum, for example, cerebrospinal infusion of Il6 is sufficient to cause radial glial cell proliferation (Shimizu et al., 2021).

To understand regenerative neurogenesis, it will be important to determine whether specific signalling molecules are sufficient to induce proliferation and/or to reprogramme stem/progenitor cells for neurogenesis, or whether precise temporal regulation of synergising signals is necessary for successful regeneration. These signals probably differ for distinct populations of stem/progenitor cells.

### Epigenetic modifiers

Epigenetic modifications (such as histone modifications that regulate transcriptional accessibility of many genes simultaneously) need to change for regenerative neurogenesis to occur (Goldman and Poss, 2020; VandenBosch and Reh, 2020; Zhang et al., 2020b). In the injured zebrafish retina, histone deacetylase 1 (Hdac1) is necessary for Müller glia proliferation by repressing the expression of Notch target gene *her4.1* (Mitra et al., 2018). Inhibition of Hdac1 using a dominant-negative, cell type-specific approach inhibits neurogenesis in uninjured and lesioned spinal cords of larval zebrafish (Cavone et al., 2021). Conversely, pharmacological inhibition of the histone acetyltransferase Ep300 inhibits expression of Notch target genes *her4* and *her6*, as well as increasing proliferation of radial glia in the optic tectum, but inhibiting neuronal differentiation (Shimizu and Kawasaki, 2021). The methylation state of genes is also important; knockdown of the injury-induced cytidine deaminase (encoded by *apobec2*) reduces Müller glia proliferation and neurogenesis (Powell et al., 2012). These examples suggest that experimentally altering epigenetic marks could be a strategy to promote regenerative neurogenesis.

### Effector genes

These signalling pathways and epigenetic modifications must converge on transcription factors that can reprogramme stem/progenitor cells for neurogenesis (Christen et al., 2010). For example, *sox2* (an important pluripotency factor) is upregulated after injury in the zebrafish CNS. Its overexpression stimulates Müller cell proliferation in the retina (Gorsuch et al., 2017) and knockdown impairs regenerative neurogenesis in the spinal cord (Ogai et al., 2014). Similarly, upregulation of the neurogenic transcription factor *achaete-scute homolog 1* (*ascl1*) is necessary for regeneration in the retina. Overexpression of *ascl1* in combination with *lin28a* overexpression and Notch suppression induces proliferation of Müller glia in the uninjured retina (Elsaeidi et al., 2018; Fausett et al., 2008). Another important gene in regenerative neurogenesis is *oligodendrocyte transcription factor 2* (*olig2*) (Reimer et al., 2009), which controls motor neuron generation in the spinal cord and is directly regulated by the Shh pathway in development (Takebayashi et al., 2002). Interestingly, *gata3* is necessary specifically for regenerative neurogenesis in the telencephalon, but not for constitutive neurogenesis (Kizil et al., 2012; Kyritsis et al., 2012). This further supports the presence of regeneration-specific intracellular signalling. It will be interesting to study in detail how the signalling pathways described above contribute to the regulation of such ‘hub genes’ across different progenitor types.

## Relevance for non-regenerating vertebrates

Although many of the regenerative signalling molecules are shared between different neural tissues, different cell types may react to an injury and alter the injury site environment. For example, the spinal cord may show a more dramatic invasion of neutrophils and fibroblasts than other CNS regions (Tsarouchas et al., 2018; White et al., 2017) and internal states of stem/progenitor cells may also differ between CNS regions, for example, as discussed above for Notch activity. However, systematic comparisons between regenerating and non-regenerating vertebrates show that effector genes of successful regeneration in zebrafish can be effectively manipulated in mammals.

Recently, single-cell RNA sequencing (scRNA-seq), as well as single-cell assay for transposase-accessible chromatin using sequencing (scATACseq) have been used to determine gene regulatory networks that are associated with regenerative ability – or the lack thereof – in the regenerating zebrafish retina, chick retina (limited regenerative potential) and non-regenerating mouse retina (Hoang et al., 2020). The study has shown that deletion of nuclear factor I genes in mice leads to increased expression of neurogenic genes, such as *Ascl1*, and generation of different retinal neuronal cell types after injury. Overexpression of *Ascl1* alone also induces limited retinal neurogenesis in mice (Jorstad et al., 2017) and overexpression in the injured mouse retina in combination with pharmacological inhibition of histone deacetylases or overexpression of the neurogenic transcription factor *Atoh7* leads to more efficient retinal neurogenesis (Jorstad et al., 2020; Todd et al., 2021).

Overexpression of the transcription factor *Olig2* in the adult mouse spinal cord through viral transduction reprogrammes spinal ependymal cells to generate oligodendrocytes instead of scar-forming astrocytes (Llorens-Bobadilla et al., 2020). These studies demonstrate that reprogramming of stem/progenitor cells is possible in the CNS of non-regenerating species. Interestingly, ventricular stem/progenitor cells may not be the only cells in the CNS that can be reprogrammed to become neurogenic (reviewed by Leaman et al., 2022). For example, somatic ‘NG2 glia’ in the spinal cord of mice can be reprogrammed by overexpression of the neural pluripotency factor *Sox2*, which leads to neurogenesis and functional improvement after spinal injury (Tai et al., 2021). Knowledge of how extracellular signalling affects the expression of neurogenic hub genes in successful regeneration will allow more targeted intervention in non-regenerating systems in the future.

## Future perspectives

Current scRNA-seq methods already give us unprecedented detail on potential signals and downstream genes in the injured zebrafish CNS. Using ‘pseudotime’ approaches, the differentiation trajectories of newly generated cells can be resolved under different experimental conditions to identify crucial steps in neuronal differentiation after injury. (Cavone et al., 2021; Cosacak et al., 2019; D'Gama et al., 2021; Klatt Shaw et al., 2021; Lange et al., 2020).

Additional techniques, such as scATACseq, to determine the accessibility of genes (Avagyan et al., 2021), or barcoding of cell clones (Raj et al., 2020), will allow us to map the intracellular pathways involved in regenerative neurogenesis in more detail. The zebrafish is particularly suited to genetic approaches; it is possible to use cell type-specific manipulations, for example by expressing a dominant-negative receptor or by cell type-specific overexpression of signalling pathway genes (Cavone et al., 2021; Lee et al., 2020). Moreover, cell type-specific approaches to gene deletion using CRISPR/Cas9 technology are becoming increasingly available (Hans et al., 2021; Li et al., 2019). These approaches could also be used to increase transcription of targeted genes through CRISPR activation (CRISPRa), or knockdown via CRISPR interference (CRISPRi) (Kampmann, 2020; Liu et al., 2019). These cell type-specific approaches will be invaluable in distinguishing between modulating global cell interactions that have a net outcome in stem/progenitor cell behaviour or direct action on stem/progenitor cells.

Repair is only achieved if appropriate cell types are produced in sufficient numbers and mature and integrate into the existing, but damaged, neuronal network. Genetic lineage tracing of stem/progenitor cell progeny will allow us to follow the differentiation and maturation trajectories of neurons from different stem/progenitor cell populations. Using these data from the regeneration-competent zebrafish in combination with single-cell omics, we can find new ways to tailor the identity of new neurons to be competent to repair an injury or replace specific cell types in degenerative conditions.
